# Letter from Paris

**Published:** 1842-10-08

**Authors:** 


### FOREIGN CORRESPONDENCE.

PARIS, September, 1842.

The most important occurrences that have lately taken place in Paris, likely to prove interesting to a professional reader, may be communicated in a few lines; hence I shall not occupy much of your valuable space with what may, after all, prove acceptable to only a limited number of your subscribers.

The Academy of Sciences has been for some time occupied with the question of the mode of formation of the caducous membrane. The discussion has been carried on, principally, by Messrs. Coste, Lesauvage, and Martin St. Ange. M. Lesauvage is of opinion, that at the period of conception, a quantity of fluid, sufficient to fill its cavity, is effused into the uterus, and that the caducous membrane is the result of a species of crystallization of this liquid; whilst M. Coste denies, altogether, the existence of the fluid, at the period when the membrane is formed, and attributes its origin to other causes, to be indicated by him at a future period. And again, in the matter of the circulation, Lesauvage admits that vessels, independent of those of the womb, are formed in the membrane itself; whilst Coste thinks that the preparations which he has exhibited to the Academy, go to prove conclusively, that the vessels seen in the caducous membrane are only prolongations of those belonging to the uterus. M. Martin St. Ange promises soon to give the result of the experiments in which he has been for a long time engaged, with the view of elucidating this interesting subject, and in the mean time requests a suspension of the discussion.

At the Royal Academy of Medicine, two elections have lately taken place. The fortunate candidates are MM. Jules Guérin, and Poiseuille.

A proposition was made to the Academy, a short time since, by the celebrated Louis, to recommend to the Government the appointment of a certain number of travelling Physicians, whose duty should be to make annual excursions into the different countries of Europe and America, occasionally extending them to Asia and Africa, for the purpose of studying on the spot such diseases as may be endemic in those countries; ascertaining the various methods of treatment pursued by the native physicians, and reporting, at convenient periods, the results of their investigations. The plan has, as yet, only been proposed to the Academy, and a committee appointed to report on its feasibility; the great popularity and influence of M. Louis are sufficient, however, to outweigh any objections that may be made by the gentlemen constituting the committee, and hence there is little doubt but that the recommendation will be made to Government, in which case it will undoubtedly be approved, and appropriations made to meet the necessary expenses of the undertaking.

Another operation of Cesarean Section has been lately performed in the Capital, by one of the most distinguished Surgeon-Accoucheurs of the day; the case was one extremely favourable for a successful termination, and the operation was well performed, and at a proper period. The issue, however, was unfortunate for the mother, who died of consecutive peritonitis on the eighth day after the operation. The infant survived, and was well six weeks after being removed.

An angry correspondence has just taken place between Monsieur Ganal, the celebrated embalmer, and Doctor Pasquier, surgeon to the late Duke of Orleans, relative to the manner in which the body of the Duke was embalmed. I gather from the letters which have been sent me by the parties, that the dispute arose in consequence of the refusal of Dr. Pasquier to employ M. Gannal to perform the operation, preferring to do it himself, after the old method, with corrosive sublimate, spices, etc., which he styles Egyptian, and which he considers better than the modern process of M. Gannal. As is usual in such cases, the terms *Charlatan*, *Voleur*, etc. etc., are of frequent occurrence, in the correspondence alluded to, and the principal object of both parties seems to be to gain notoriety.

Death has been busy of late amongst the savans of our profession. Monsieur Double, quite a distinguished physician, and known favourably for his devotion to his profession, which led him, some two years since, to refuse a Peerage, which was offered to him on the condition of his relinquishing the practice of medicine, was the first of four of his contemporaries to be called to his last account. His death was occasioned by pulmonary apoplexy, and hastened by his obstinacy in refusing to call in medical advice; continuing to treat himself for another disease, until the last two or three days, when his friends positively insisted on the attendance of M. Andral. M. Double was not an author of any note, but in the enjoyment of a large and lucrative practice, and closely allied with many of the most distinguished characters of the day. It was at the house of Marshal Soult, and whilst seated in the garden, between the Marshal and his lady, that he experienced the first attack of the disease which terminated in his death.

Monsieur Edwards, the distinguished zoologist, and member of the two Academies of Sciences and Medicine, died at Versailles, within a week or two after the death of Dr. Double.

The celebrated chemist, Pelletier, a professor in the Paris School of Pharmacy, and likewise member of the leading Academies of Paris, died on the 22d July, much regretted by all who knew him. It was to him, in conjunction with Caventou, that the profession, and community at large, owed a lasting debt of gratitude, for his experiments on the vegetable alkalies and the discovery of quinine.

The immortal Larrey was the last to feel the stroke of the dread leveller. He died at Lyons, on the 25th of July, on his return from Algiers, where he had been sent by the minister of war, to make a medical inspection of the army. Fortunately, his son had accompanied him, and was present to render the last services to his distinguished father. The body was transported to Paris, and interred at Père La Chaise, where the city had gratuitously furnished a vault for its reception, with considerable pomp and ceremony. Eloquent eulogies were pronounced over the tomb, by Breschet, in the name of the Institute; Pariset, its perpetual Secretary, for the Royal Academy of Medicine; Michael Levy, in the name of the Professors of the military school and hospital of Val-de-Grace; Baudens, in that of the French Army, and Guyon, for the Army of Africa. Military honours followed; and the scene closed over the great Surgeon of the Armies of the Empire, one of the most devoted followers and friends of Buonaparte. It would require too much space, to trace here a biographical notice of this Chief of Military Surgeons. His life was so eventful a one, that many pages would be required for that purpose. He has left works behind him, however, which must always survive, and the name of Larrey will go down to posterity coupled with that of his friend and benefactor, the great Emperor.

After a most brilliant concours, an election has been made to the Chair of Clinical Surgery, in the Faculty of Medicine, vacated by the death of Sanson the elder. There were eleven candidates for the professorship, and all of them men of merit—some of distinction—so that it is no small eulogy on the one selected,—Bérard, the younger,—to say that amongst his competitors were Malgaigne, Vidal de Casis, Laugier, and Robert. Each candidate acquitted himself in a manner highly satisfactory to the Judges and to the public, who assisted at the different examinations, and it was thought to be a difficult task for the committee to make their selection. Their unanimity, however, shows that they estimated at their proper value the talents of the gentleman on whom their choice fell, and his peculiar qualifications for a lecturer. Great applause followed the announcement of the success of M. Bérard, in the Royal Academy of Medicine, of which he is one of the most popular members. The vote stood—

| | |
|---|---|
| For Bérard, - - - - - - | 8 |
| Vidal de Cassis, - - - - - - | 2 |
| Laugier, - - - - - - | 1 |
| Robert, - - - - - - | 1 |

There has been for the last three months, an epidemic typhoid fever, prevalent at Paris, which although mild in its nature, and rarely proving fatal, was very general, and often tedious in its course. It was attributed to the uncommon heat and dryness of the season, and has been on the decline, since the occurrence of a change in the weather.

